# Immunocytochemical Characterization of Alzheimer’s Disease Hallmarks in APP/PS1 Transgenic Mice Treated with a New Anti-Amyloid-*β* Vaccine

**DOI:** 10.5195/cajgh.2013.119

**Published:** 2014-03-27

**Authors:** Ivan Carrera, Ignacio Etcheverria, Yi Li, Lucia Fernandez-Novoa, Valter Lombardi, Carmen Vigo, Hector H. Palacios, Valery V. Benberin, Ramon Cacabelos, Gjumrakch Aliev

**Affiliations:** 1 Department of Neurosciences, EuroEspes Biotechnology, La Coruna, Spain; 2 Yale University School of Medicine, New Haven, CT; 3 Atlas Pharmaceuticals, Sunnyvale, CA; 4 National Institute on Aging, National Institutes of Health, Baltimore, MD; 5 Medical Center of the Administration of the President of the Republic of Kazakhstan, Astana, Kazakhstan; 6 EuroEspes Biomedical Research Center, Institute for CNS Disorders and Genomic Medicine, La Coruna, Spain; 7 GALLY International Biomedical Research Consulting LLC, San Antonio, TX; 8 Department of Health Science and Healthcare Administration, University of Atlanta, Atlanta, GA

**Keywords:** Alzheimer’s disease, anti-amyloid-β vaccine

## Abstract

**Introduction:**

APP/PS1 double-transgenic mouse models of Alzheimer’s disease (AD), which overexpress mutated forms of the gene for the human amyloid precursor protein (APP) and presenilin 1 (PS1), have provided robust neuropathological hallmarks of an AD-like pattern at early ages. This study aimed to characterize immunocytochemical patterns of the AD mouse brain, which is treated with the EB101 vaccine, as a model for human AD.

**Material and methods:**

In this novel vaccine, a new approach has been taken to circumvent past failures with A*β* vaccines by judiciously selecting an adjuvant consisting of a physiological matrix embedded in liposomes, composed of naturally occurring phospholipids (phosphatidylcholine, phosphatidylglycerol, and cholesterol).

**Results:**

Our findings showed that the administration of amyloid-*β*1–42 (A*β*) and sphingosine-1-phosphate emulsified in liposome complex (EB101) to APP/PS1 mice before the onset of A*β* brain deposition (at 7 weeks of age) and/or at an older age (35 weeks of age) can be effective in both halting the progression and clearing the AD-like neuropathological hallmarks. In addition, passive immunization with EB101 did not activate inflammatory responses from the immune system and astrocytes. Consistent with a decreased inflammatory background, the basal immunological interaction between the T cells and the affected areas (hippocampus) in the brain of treated mice was notably reduced.

**Conclusion:**

These results provide strong evidence that immunization with the EB101 vaccine prevents and attenuates AD neuropathology in this type of double-transgenic mice.

